# Anti-Reflective Zeolite Coating for Implantable Bioelectronic Devices

**DOI:** 10.3390/bioengineering9080404

**Published:** 2022-08-20

**Authors:** Giuseppe Oliva, Maria Giovanna Bianco, Antonino S. Fiorillo, Salvatore A. Pullano

**Affiliations:** 1 BATS Laboratory, Department of Health Sciences, Magna Græcia University of Catanzaro, Viale Europa, 88100 Catanzaro, Italy; 2 Department of Medical and Surgical Sciences, Magna Græcia University of Catanzaro, Viale Europa, 88100 Catanzaro, Italy

**Keywords:** implantable device, medical application, nanoporous material, anti-reflective coating, solar cell

## Abstract

Since sunlight is one of the most easily available and clean energy supplies, solar cell development and the improvement of its conversion efficiency represent a highly interesting topic. Superficial light reflection is one of the limiting factors of the photovoltaic cells (PV) efficiency. To this end, interfacial layer with anti-reflective properties reduces this phenomenon, improving the energy potentially available for transduction. Nanoporous materials, because of the correlation between the refractive index and the porosity, allow low reflection, improving light transmission through the coating. In this work, anti-reflective coatings (ARCs) deposited on commercial PV cells, which were fabricated using two different Linde Type A (LTA) zeolites (type 3A and 4A), have been investigated. The proposed technique allows an easier deposition of a zeolite-based mixture, avoiding the use of chemicals and elevated temperature calcination processes. Results using radiation in the range 470–610 nm evidenced substantial enhancement of the fill factor, with maximum achieved values of over 40%. At 590 and 610 nm, which are the most interesting bands for implantable devices, FF is improved, with a maximum of 22% and 10%, respectively. ARCs differences are mostly related to the morphology of the zeolite powder used, which resulted in thicker and rougher coatings using zeolite 3A. The proposed approach allows a simple and reliable deposition technique, which can be of interest for implantable medical devices.

## 1. Introduction

In the last decade, research has driven a series of technological innovations in the fields with even more increasing energy demand [1,2,3,4]. This is evident in industrial technologies characterized by huge energy consumption (e.g., healthcare, automotive, consumer electronics, industrial, etc.) [5,6]. In healthcare modern diagnostic approaches and therapies, implantable bioelectronic devices can improve the clinical outcome of the patient, obtaining a large amount of data [7,8,9]. Innovative devices are usually light, allowing the patient to conduct normal daily activities [10,11,12]. Energy storage is by far one of the bottlenecks, preventing efficient energy management [13]. The latest designed batteries could even last for years, also thanks to energy harvesting techniques that collect energy from external sources [14,15]. Thus, the evolution of medical electronic devices is aimed at the reduction of energy consumption (in the order of μW or less) and the simultaneous harvesting of energy from the environment (e.g., solar cells, piezoelectric generators, triboelectric generators, radio frequency harvesters, thermo-electric conversion, etc.) [14,15,16,17,18,19]. In comparison, solar cell technology is characterized by a higher energy density coming from photoelectric transduction and is more effective for energy harvesters [3,4,20]. Even just a few minutes of direct sunlight exposure of the subcutaneous implanted solar cells can provide enough energy to an implantable medical device for an entire day [21,22]. The main challenge in developing solar-powered systems for implantable subcutaneous medical devices is being able to ensure a regular power supply, even in low-light conditions [21]. Obviously, not all solar radiation is converted into electricity. Silicon solar cells have a value that slightly exceeds 20%, while more recent single-junction silicon solar cells reach an efficiency of up to 28% [23]. Perovskite solar cells achieved an efficiency level of up to 25.8%, while monolithic perovskite/silicon tandem solar cells reached an efficiency level greater than 29% [24,25]. Some of the factors that limit the conversion efficiency are the reflection of incoming radiation, photon energy, carrier recombination and parasitic dissipative elements of the PV cells [23,26]. To minimize the reflection of the incident light, various technologies, such as the deposition of an anti-reflective coating (ARC), have been investigated for reducing the surface reflectance close to 1% [27]. ARC is designed as a thin film, having a thickness that ranges from a minimum of a few tens of nanometers up to a few micrometers [28]. They are designed and fabricated according to the solar spectrum, showing a higher performance in the wavelength range from 300 nm to 24,000 nm [29]. To this end, different kinds of materials and techniques (single layer, multilayers, or gradient refractive index nanostructures), have been investigated [30]. Silica-based materials, such as SiO_2_ and SiN and silicon-based nanostructures (i.e., nanowires, nanopillars, nano cones, etc.) have found wide application in PV cells as anti-reflective coatings [30,31,32]. Another class of materials with interesting anti-reflective properties is that of metal oxide (e.g., TiO_x_, Ta_2_O_5_, ZnS, Al_2_O_3_, MgF_2_) or a combination of these materials [33,34]. They are characterized by a well-matched refractive index and high mechanical resistance together with low deposition cost [34]. More recently, anti-reflective coatings made of polymeric materials were proposed as valid alternatives with inherently self-cleaning properties [35,36,37]. Nanoporous materials were recently investigated as anti-reflective coatings because of the correlation between the refractive index and the porosity, improving light transmission. The availability of materials with a low refractive index is limited, therefore porous materials are used to lower and obtain the required refractive index by controlling the density of the material [38,39,40]. Zeolite constitutes a family of mineral aluminum-silicates, structurally different from normal silicates or aluminates and widely used in ion exchange applications and molecular sieves. They possess well-determined porosity and a low refractive index [39,40,41]. An important aspect of the use of materials for implantable devices is biocompatibility, which is related to the interaction between the outer layer and the tissues/cells. The latter should avoid cytotoxicity or promoting immunological rejection [42,43]. As reported in literature, several types of zeolites, often in the form of composites, were investigated as biocompatible material for biomedical applications (e.g., in tissue engineering, drug delivery systems, wound healing, etc.) [44,45,46,47]. In this work, two types of synthetic zeolite that are part of the Linde Type A (LTA) family, specifically type 3A and 4A, having a different grain size and a slightly different pore dimension, were investigated. An easy deposition technique onto commercial PV cells for the fabrication of anti-reflective coatings was then investigated. ARCs were analyzed, using white light and monochromatic radiation. It was experimentally observed how the specific nanoporous material affects the performance of the solar cell. The work is thus focused on providing insights on the deposition of a zeolite-based ARC and its spectral behavior for the design of optimized implantable cells.

## 2. Materials and Methods

**Figure 1:**
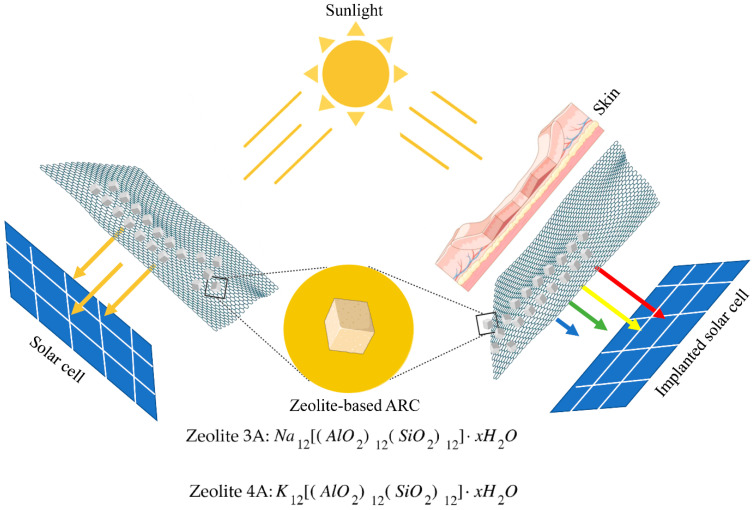
Schematic representation of the zeolite-based anti-reflective coating and the chemical formula of the zeolite 3A and 4A employed during the preparation of the mixtures.

The interaction of photons with skin can be considered by different points of view; physical (depth of penetration), chemical (absorption of light and subsequent photochemical events) and biological (tissue/cell response). In the human skin, the depth of penetration increases as the wavelength increases. Red light is the most penetrating (about 4–5 mm), while blue light penetrates less (about 1 mm) and UV light hardly penetrates under the skin (about 0.5 mm). This is because the skin has a wide range of natural substances, called chromophores (e.g., water, hemoglobin, melanin), where each of them can absorb specific wavelengths [48]. A schematic representation of the proposed concept, together with the chemical formula of both types of zeolites, is reported in Figure 1. Regarding the standard working model of a PV cell in which solar radiation directly hits the surface (Figure 1a), in case of applications that require the implantation of the energy source (Figure 1b), it is important to evaluate the specific wavelength, its intensity and the depth of penetration for an optimized optoelectric transduction.

To this end, a commercial, bare solar cell made of poly-Si with a dimension of 52 × 26 mm and a thickness of 250 μm was used. Rated transduction efficiency for this kind of industrial cell is about 15% with a maximum voltage of 0.52 V and a maximum current of 0.49 A (maximum power 0.253 W). The ARC deposition was performed by spin-coating using a mixture of zeolite powder and vegetable oil [31,32]. Zeolite LTA powders of type 3A and 4A were used. Zeolite 3A (UOP-Honeywell, Reggio Calabria, Italy) is a K form of type A with the pore size of 3.2 Å, while zeolite 4A (Luoyang Jianlong Micro-nano New Materials Co., Ltd., Yanshi, China) is the Na form, characterized by openings of 4.2 Å. Zeolite 3A was provided in fine-grained powder with cubical particles of 2 μm in size (average) and a bulk density of 848 kg/m^3^. Zeolite 4A is characterized by cubical particles of 450 nm in size (average) and a bulk density of 770 kg/m^3^. The Si/Al ratio for both types is close to 1.00. A refined soybean vegetable oil, composed of 54.4% of 9,12-octadecadienoic acid (55.4%), 26.3% of 9-octadecenoic acid, 12.8% of hexadecanoic acid, 4.2% of octadecanoic acid and 1.3% of 12-octadecenoic acid, was used. The oil is characterized by density of 914 kg/m^3^, typical viscosity of 33 cSt at 35 °C, a smoke point of 232 °C, a flashpoint of 254 °C and an iodine value of 143. The composition of the mixtures is expected to influence the thickness of the layer and the superficial concentration. Mixtures were prepared using a homogenizer (IKA T 25 Ultra Turrax, Staufen im Breisgau, Germany) at 25,000 rpm using a percentage of 60% *w*/*w* of zeolite and 40% *w*/*w* of vegetable oil, then they were sonicated (Shesto UT8031, Watford, UK) for 15 min (40 kHz, 100 W). Subsequently, the mixtures were spun onto the PV cell at different rotational speeds (from 2000 up to 6000 rpm for 60 s) then annealed at 200 °C for about 3 h. The annealing process depends on the composition of the oil used, chosen among those having high iodine (parameter based on the reactivity of alkyl double). This results in lower temperature decomposition of oil (greater number of double bonds), which acts as a dried supporting matrix for the ARC.

Morphology of the layer was analyzed through scanning electron microscopy (Carl Zeiss EVO HD15, Jena, Germany) and profilometry (Veeco Dektak 6M, New York, NY, USA). The electrical characterization of solar cells was performed using both unpolarized white light (400–750 nm) and monochromatic light radiation in the visible range (470–610 nm) to reveal differences in the photoelectrical transduction. To emulate the initial part of the solar spectrum, a battery of light-emitting diode (LED) was used, with a wavelength of 470 nm (blue), 530 nm (green), 590 nm (yellow) and 610 nm (red). Characteristics of the single LEDs are reported in Table 1.

The unpolarized white light was placed perpendicular to the PV cell, biasing the LEDs according to the manufacturer’s specification (obtaining the maximum brightness). PV cells were electrically monitored using an electrometer (Keithley Instruments 6517B, Cleveland, OH, USA) and a voltmeter (Fluke Corporation 8808A, Washington, DC, USA) and the fill factor (FF) was then evaluated. FF depends on the values of current and voltage as follows:

(1) $$FF=\frac{P_{m}}{V_{oc}\cdot I_{sc}}=\frac{V_{m}\cdot I_{m}}{V_{oc}\cdot I_{sc}}$$

which is the ratio between the maximum power (*P_m_*) and the open circuit voltage (*V_oc_*) multiplied by the short circuit current (*I_sc_*) [49]. Finally, we investigated the emissive characteristics of the coated solar cell since a basic requirement for a biomaterial is the absence of any emission of potentially dangerous substances as a result of soybean thermal oxidation after annealing. To detect any emissions of substances from the coated solar cell, a photoionizer (MiniRae 3000 PID) was used to evaluate emission patterns during the final part of the annealing process. The technique is based on gas ionization by ultraviolet (UV) light with a wavelength of 120 nm and energy of 10.6 eV.

## 3. Results

**Figure 2:**
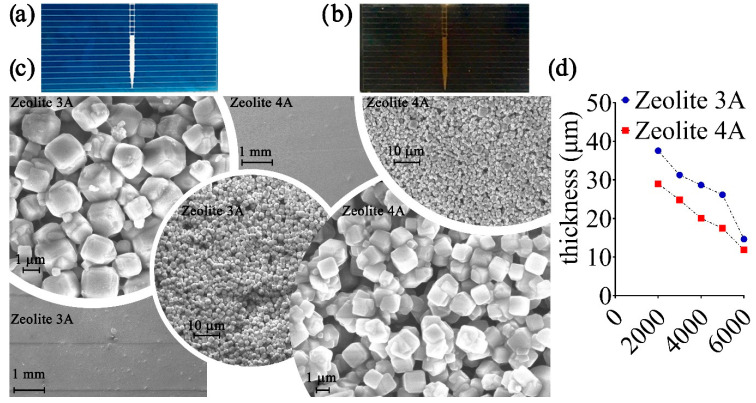
(**a**) Standard bare solar cell and (**b**) solar cell with anti-reflective coating. (**c**) SEM images of deposited zeolite 3A and zeolite 4A which show substantial similarity in terms of grain geometry despite the different dimensions which are smaller (about 450 nm) for type 4A as compared to type 3A (about 2 μm). (**d**) Thickness of the ARCs vs. deposition speed.

To compare the results, we used control solar cells, which have no type of anti-reflective film deposited on them (with and without thermal annealing process). The characterization that concerns the experimentation involves twelve PV cells, two of which are control cells (with and without 200 °C thermal annealing process), five cells with zeolite 3A coatings and five cells with zeolite 4A coatings. SEM analysis of Figure 2 evidenced the composition of zeolite 3A and 4A and the morphological characteristics of the anti-reflective coating at different magnifications.

**Figure 3:**
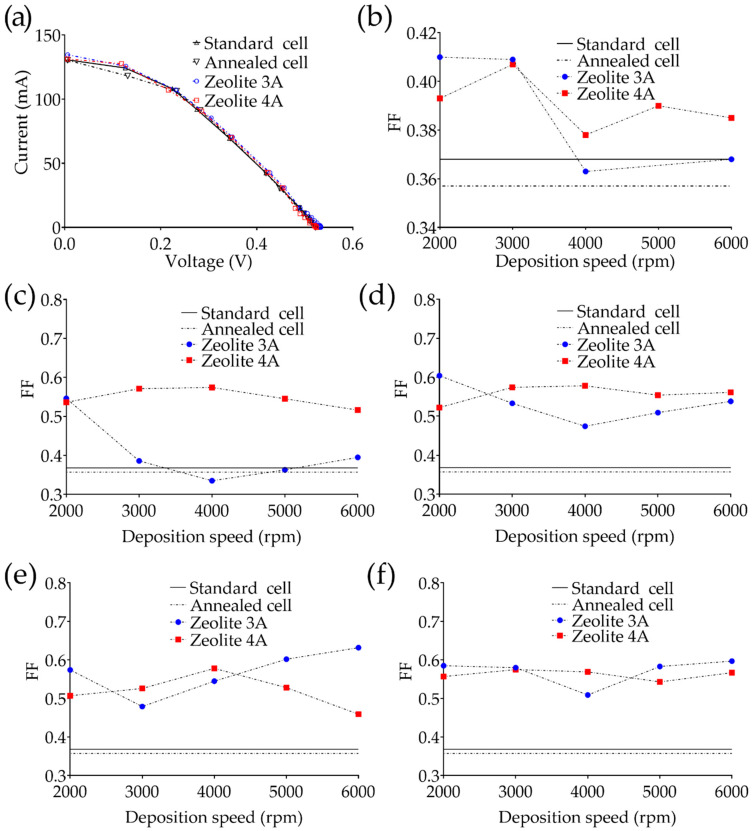
(**a**) I-V characteristics of: standard, annealed solar cell and two representative cells coated with zeolite type 3A and 4A. Fill factor vs. deposition rate in the range 2000–6000 rpm using (**b**) unpolarized white light, (**c**) blue light at 470 nm, (**d**) green light at 530 nm, (**e**) yellow light at 590 nm and (**f**) red light at 610 nm.

As evidenced also in Figure 2d, the thicknesses of the deposited layer range from 37.43 µm down to 14.65 µm for the zeolite-3A-based ARC and from 28.98 µm down to 11.86 µm for the zeolite-4A-based ARCs obtained by increasing the deposition speed from 2000 up to 6000 rpm. Subsequently, both types of deposited ARC were analyzed by means of a profilometric analysis evidencing surface roughness of about 1.51 ± 0.06 µm for type-3A-based samples and 0.47 ± 0.02 µm for type 4A layers. The FF depends on the maximum power of the solar cell, to the *I_sc_* and *V_oc_*, according to Equation (1). I-V characteristics and FF were estimated using white light and then the most significant subcomponents as reported in Figure 3a in which bare solar cells are compared with two representative samples coated with zeolite 3A and 4A. All light sources were placed at 0.1 m with a collimated and non-polarized beam.

**Figure 4:**
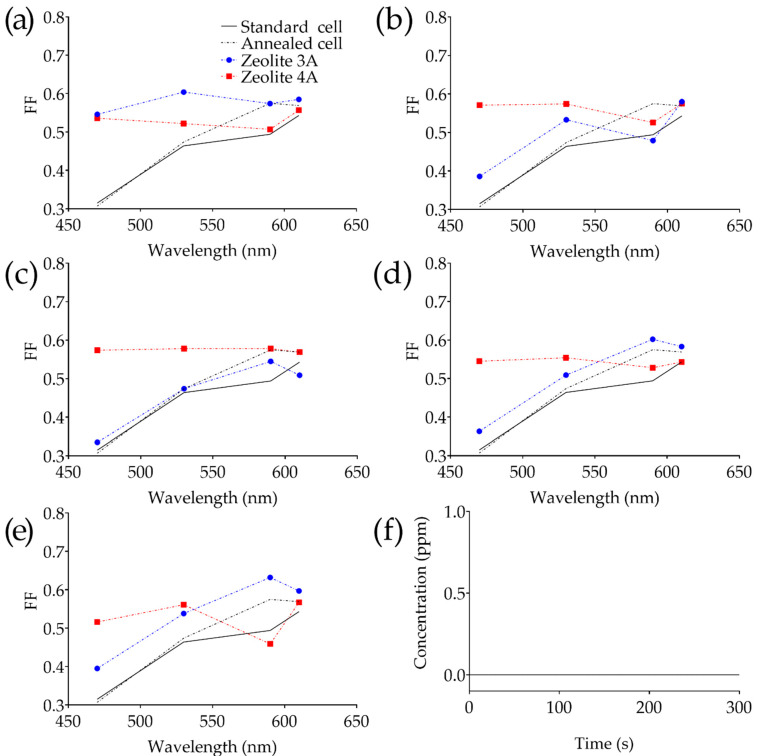
Fill factor vs. wavelength for anti-reflective layer deposited at (**a**) 2000 rpm, (**b**) 3000 rpm, (**c**) 4000 rpm, (**d**) 5000 rpm and (**e**) 6000 rpm. (**f**) Representative emission profiles of solar cells coated with zeolite 3A and 4A at 2000 rpm.

Furthermore, for each solar cell used for the experiment, the relative I-V characteristics were evaluated at the different wavelengths reported in Table 1. The brightness of the light source was evaluated through a luxmeter, and all the acquisitions were made under the same environmental conditions. The fill factor, as reported in Figure 4a–e, was also evaluated with respect to the wavelength to highlight the spectral behavior of each ARC. Finally, at the end of the annealing process, the coated cell was monitored for 300 s to evaluate the emission of substances from the nanoporous layer (Figure 4f).

Since the light intensity was variable depending on the specific wavelength reported in Table 1, transduction efficiency using three different intensities was evaluated to investigate its effect on the FF. The light at 470 nm was varied in the range 21,000–74,000 lux, the light at 530 nm in the range 15,000–46,000 lux, the light at 590 in the range 1800–9000 and the light at 610 nm in the range 4600–15,000 lux. The relative variation in the FF was of 2.2% (470 nm), 1.4% (530 nm), 3.8% (590 nm) and 0.1% at 610 nm.

## 4. Discussion

In the biomedical field, the growing development of implantable electronic devices is boosted by development of a specific technology for energy harvesting (e.g., solar cell technology). Specifically, they can absorb light transmitted through the skin in the visible and near-infrared band, to self-recharge and power-up subdermal medical devices [50]. They should be characterized by an elevated level of safety, light weight, biocompatibility, reduced dimensions and conformability. The joint use of a subdermal medical device and energy harvesting technology (e.g., solar cell) can enable continuous or on-demand monitoring of physiological parameters on patients by eliminating/reducing, periodic battery replacement (e.g., pacemakers, glucose level monitors, etc.) [43,45]. Any ARC aims to achieve zero reflectance. In the case of a single ARC, it can be achieved when the product of the refractive index of air (n_air_) and that of the substrate (n_s_) equals the square of the anti-reflective coating (n_ARC_), and thus n_air_·n_s_ = n_ARC_^2^. Since n_air_ ≃ 1, the refractive index of the ARC depends on √n_s_ [28]. A material in which the refractive index is reduced along with its thickness, or a porous material, represents a strategy for the fabrication of anti-reflective coatings. Well-defined surface porosity results in an overall refractive index that depends on the imparted porous topology. The latter depends on the percentage of porosity, its thickness and the refractive index of the material itself [50]. Concerning other porous materials investigated as ARCs (i.e., porous silicon), zeolite is inherently characterized by a well-defined and controllable porosity during its synthesis [28,51]. If the dimensions are carefully chosen to be smaller, if compared with the wavelength, the light becomes trapped inside the film [39,40]. Since the refractive index of zeolite 3A and 4A is less than one, it is expected to approach the zero-reflectance condition for the proper deposition of a zeolite layer [52]. Moreover, recent literature evidenced that zeolite, due to its hydrophilic properties, transparency and scratch resistance, is a promising candidate for AR coating. However, one of the key issues is the deposition process which often involves in situ and seeded growth, requiring synthesis of the layer using chemical solutions (most of them are highly basic), hindering the deposition of silica-based substrate [53,54]. Moreover, the calcination process at temperatures higher than 400 °C for different hours is harmful for the PV cell [39,53,54]. The coating mixture prepared by vegetable oil and zeolite powder allows, after a low-temperature annealing process, the formation of a supporting matrix of the layer which is scratch-resistant and stable over months. Considering the above, a simple method which avoids direct synthesis of zeolite and thermal stress can be promising for easier deposition of AR coatings. Another important aspect summarized in Figure 1b is the operating regime of devices involving the use of implanted PV cells. Let us consider the skin as a complex organ that has evolved as the outer interface between the body and the environment [55]. It is composed of a layered structure (i.e., epidermis, dermis, subcutaneous tissue, etc.), each one possessing a peculiar composition and functions. For example, the epidermis is a natural filter for ultraviolet radiation, but at the same time, the latter is one of the major sources which trigger vitamin D production [45,46]. Similarly, while about 50% of the light intensity is reflected, scattered and absorbed on the external skin layer, the different wavelengths penetrate differently into the skin. As the wavelength increases from blue to red, the depth of penetration increases accordingly (typically from about 1 mm to 5 mm ore [56]. Thus, it is expected that implanted PV cells should be stimulated by visible light with a wavelength in the red region.

Both types of layers, as shown in Figure 2b, appear as a clear film with a slight yellowish color which depends on the use of vegetable oil. Even though the morphology of both anti-reflective proposed coatings is similar (Figure 2), the geometrical characteristics (e.g., thickness, roughness) between them are different because of the different grain size. The latter, as expected, influences the characteristics of the anti-reflective coating. Regarding the solar cells on which an anti-reflective coating characterized by zeolite 3A and 4A has been deposited, the surface of the coating has a rough consistency, due to the specific material used, which is comparable to one of the bare PV cells. The nanoporous structure refers to the atomic level, and the cavities have typical dimensions much lower than the wavelength of the sunlight. This is an advantage for solar applications, as the light is mostly confined inside the layer, reducing the reflection [28,51]. At the same time, it is expected that the increased quantity of light gives rise to improved output performances of the PV cells. As the rotational speed increases, considering the same annealing process, ARCs showed progressive worsening of uniformity, with an average thickness reported in Figure 2d.

Comparing all the anti-reflective coatings, it can be observed that those based on zeolite 4A have a more uniform composition, and lower thickness and roughness. Only at a deposition speed higher than 5000 rpm did the average thickness evidence non-uniformity (PV cells not properly coated). Concerning zeolite 3A coating, worsening of the layer uniformity is evidenced as the rotation speed increases 3000 rpm. This aspect can be justified since zeolite 4A is characterized by a particle size four times lower than that of zeolite 3A. Summarizing the efficiency of each PV cell, the evidence was that it is variable depending on the wavelength, the type of zeolite and the geometrical characteristics of the layer according to Table 2.

Using unpolarized white light, it is clear how the annealing process involves a decrease of about 3% of the FF if compared with the uncoated solar cell. The deposition of zeolite-4A-based ARCs showed an FF which varies from 0.378 to 0.407, with an improvement of 12% if compared to the uncoated/annealed cell and 10% if compared to the uncoated bare cell. An improvement can also be observed in the zeolite-3A-based coatings, where the better performing cells are those coated with a lower speed (2000 and 3000 rpm for 60 s), which showed an increase in the FF of about 13% if compared to the coated/annealed cell and of about 10% if compared with the uncoated bare cell. Subsequently, since the solar spectrum is composed of specific wavelengths, which can vary in intensity depending on different parameters (e.g., position, altitude, daytime, etc.), FF at specific wavelengths was evaluated (see Table 3).

At 470 nm, the zeolite-3A-based coatings showed a significant improvement of about 42% in FF in the cell coated at 2000 rpm, with a subsequent oscillation between 6% and 20% as the rotational speed increases (thinner layer). Zeolite-4A-based coatings instead showed a notable improvement in FF from 39% at 6000 rpm, up to 45% at 4000 rpm. Considering the radiation at 530 nm, zeolite-3A-based coatings evidenced a still significant improvement in FF with a maximum of 23% at 2000 rpm. Zeolite-4A-based coating also has an almost linear trend in the green wavelength, with a maximum improvement of 20% (at 4000 rpm). In the yellow (590 nm), the improvement in FF appears to be less evident, especially if compared with the standard solar cell. Zeolite-3A-based coatings showed an improvement in FF ranging from 18% up to 22% (at 5000 and 6000 rpm). As for the cells characterized by zeolite 4A, the maximum improvement is higher than 15% at 4000 rpm. Finally, in the red region (610 nm), the improvement evidenced for the zeolite-3A-based coatings is from 7% up to 10% (at 5000 and 6000 rpm, respectively), while the 4A counterpart showed an increase from 5% and 6% (at 3000 and 4000 rpm, respectively).

**Figure 5:**
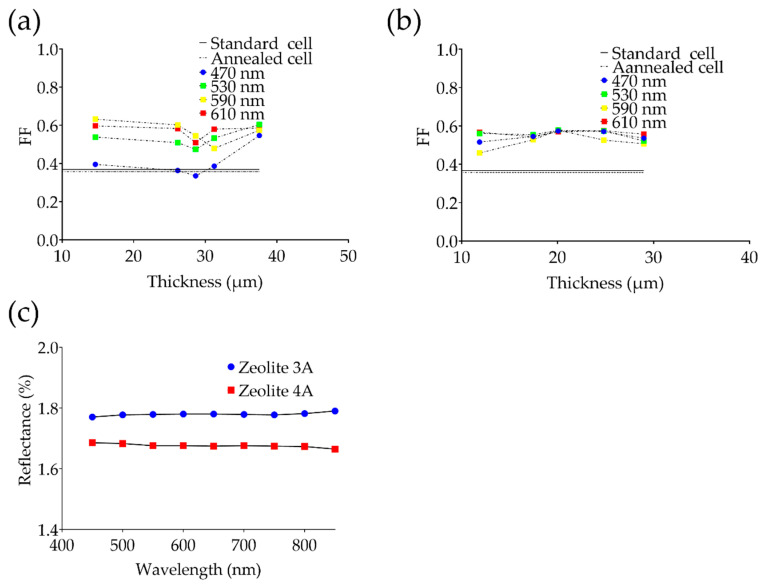
Fill factor vs. thickness for anti-reflective layer (**a**) based on zeolite 3A and (**b**) zeolite 4A. Diffuse reflectance obtained from the thinner ARCs based on zeolite 3A (thickness of 14.65 μm) and zeolite 4A (thickness of 11.86 μm) in the range 450–850 nm (**c**).

Data evidenced that, even though the deposition of the AR coatings improves the efficiency of the cells, the latter inherently decreases the efficiency as the wavelength moves toward a smaller wavelength part of the spectrum. It is interesting to note that the zeolite-3A-based coatings, characterized by grain size of about 2 μm, evidenced a significant improvement of the cell efficiency, even though the deposition results in a thicker layer (about 25% thicker than that of zeolite 4A), as reported in Figure 5a,b (the thickness is related to the deposition rate as reported in Figure 2d). Another important aspect to consider is the orientation of the powder cubical grains during the deposition process, which results in random orientation onto the PV cell. Moreover, roughness of the bare PV cell together with the presence of upper electrodes will contribute to the variability of the FF. An estimation of the spectral adsorption and reflection is given in Figure 5c, in which diffuse reflectance for two representative samples recorded through a UV-Vis spectrophotometer and an integrating sphere is shown. Data were recorded on the thinner zeolite 3A and 4A coatings, showing an average diffuse reflection of 1.68% (zeolite 4A) and 1.78% (zeolite 3A).

Literature reports different investigations on candidate AR materials for solar cell application (Table 4).

Based on the results, it was evidenced that the well-defined porosity of zeolite 3A and 4A (3.2 Å and 4.2 Å, respectively), chosen to be smaller if compared with the radiation wavelength, together with the characteristic low refractive index, allows one to approach the zero-reflectance condition so the light becomes trapped inside the film. Another aspect concerns the powder grains which mostly affect the morphology of the film. The use of fine-grained powder allows the deposition of thinner and smoother film but, conversely, a finer powder can result in a greater volume of zeolite needed and therefore a difficulty in the deposition process. As also evidenced in Figure 5c, the average diffuse reflection evidenced fairly uniform behavior of the ARC in the visible spectrum and thus the FF differences are mostly related to the specific PV cells used. Although in its initial stages, this technology appears to be interesting in order to power-up current implantable medical devices more efficiently. The spectrum of the direct solar light that affects the skin is changed in its travelling through the dermal layers. Since the NIR light easily penetrates skin, until 4–5 mm, the deposition of thin layer of ARC with zeolite onto the PV cell enhances the efficiency of converting sunlight into an easily available energy source. Attempts to integrate the PV cell into medical applications evidenced as widespread medical devices such as pacemakers or wireless communication devices might be batteryless, using sunlight energy harvesters (even indoors) [60]. Finally, an important aspect to consider is biocompatibility. Natural and synthetic zeolites were recently investigated as biocompatible and safe materials for biomedical applications, such as in wound healing and drug delivery applications as well as antibacterial agents [61,62]. Moreover, they are well known for their absorption, especially of harmful species (heavy metal ions, dyes, herbicides) [63]. In fact, the structure of the zeolite facilitates the absorption of all positively charged materials/ions, as in the case of toxic matters. Moreover, it was recently investigated as a substrate for tissue regeneration. In vitro studies showed that fibroblasts and mouse pluripotent embryonic stem cells increase their cell adhesion and proliferation on the zeolite-coated substrate [64]. Emissive analysis performed during the last step of the fabrication at 200 °C (Figure 4e) evidenced that the layer does not emit molecules in the gas phase, and this can be considered promising in a real scenario application.

## 5. Conclusions

In this work, the anti-reflective properties of zeolite-based AR coatings deposited on commercial solar cells were investigated. The proposed approach allows for an easier deposition since it rests on a spin-coating procedure and subsequent annealing. The aim is to improve the performances of the PV cell to provide energy to subdermally implantable medical devices, avoiding the use of batteries. Investigations performed on two types of zeolite powder (3A and 4A) evidenced in both cases an effective efficiency improvement, and thus the coating pointed out its inherent anti-reflective characteristics, determining a low refractive index for incoming light. Considering the higher wavelength regions (e.g., 590 and 610 nm), which are the most interesting bands for implantable devices, FF is improved by means of the proposed coatings (with a maximum of 22% and 10%, respectively). In the medium-low radiation range, the improvement of the fill factor is remarkable, with a maximum increase of more than 40%, while at medium-high wavelengths taken into consideration, the improvement reaches a maximum of 20%.

## Figures and Tables

**Table 1:** Characteristics of the light sources used to characterize solar cells coated with different zeolite layers.

Source Type	Forward Voltage (V)	Forward Current (mA)	Wavelength (nm)	h Intensity (mcd)
Blue	3.2	20	470	11,000
Green	3.2	20	530	38,000
Yellow	2.0	20	590	8000
Red	2.1	50	610	15,000

**Table 2:** Fill factor vs. deposition parameters for zeolite-coated and control solar cells.

Type	2000 rpm	3000 rpm	4000 rpm	5000 rpm	6000 rpm
FF Zeolite 3A	0.410	0.409	0.363	0.366	0.368
FF Zeolite 4A	0.393	0.407	0.378	0.390	0.385
FF Uncoated	0.368				
FF Uncoated/annealed	0.357				

**Table 3:** Fill factor vs. deposition parameters for zeolite-coated and control solar cells considering incoming radiation wavelength.

FF zeolite 3A	2000 rpm	3000 rpm	4000 rpm	5000 rpm	6000 rpm
Blue (470 nm)	0.546	0.386	0.335	0.363	0.395
Green (530 nm)	0.604	0.533	0.474	0.509	0.538
Yellow (590 nm)	0.574	0.479	0.545	0.602	0.632
Red (610 nm)	0.585	0.580	0.509	0.583	0.597
**FF zeolite 4A**					
Blue (470 nm)	0.536	0.571	0.574	0.545	0.516
Green (530 nm)	0.522	0.574	0.578	0.554	0.561
Yellow (590 nm)	0.507	0.526	0.578	0.528	0.459
Red (610 nm)	0.557	0.575	0.569	0.543	0.567
**Uncoated**					
Wavelength (nm)	470	530	590	610	
Standard Cell	0.307	0.474	0.575	0.569	
Annealed/Uncoated	0.315	0.464	0.494	0.543	

**Table 4:** Performances of anti-reflective coatings for PV cell.

ARC	PV Cell Type	ΔFF (%)	Ref.
ZnO nanorod arrays	Cu(In,Ga)Se_2_ thin film	1.3	[57]
SiO_2_/TiO_2_	Monocrystalline Silicon	6	[58]
Amorphous ZrO_x_	Silicon	1	[59]

## Data Availability

Not applicable.
